# Real-Time Loosely Coupled 3DMA GNSS/Doppler Measurements Integration Using a Graph Optimization and Its Performance Assessments in Urban Canyons of New York

**DOI:** 10.3390/s22176533

**Published:** 2022-08-30

**Authors:** Hoi-Fung Ng, Li-Ta Hsu, Max Jwo Lem Lee, Junchi Feng, Tahereh Naeimi, Mahya Beheshti, John-Ross Rizzo

**Affiliations:** 1 Department of Aeronautical and Aviation Engineering, The Hong Kong Polytechnic University, Hong Kong 999077, China; 2 Grossman School of Medicine, New York University, New York, NY 10016, USA

**Keywords:** localization, navigation, smartphone, GNSS, 3D building models

## Abstract

Smart health applications have received significant attention in recent years. Novel applications hold significant promise to overcome many of the inconveniences faced by persons with disabilities throughout daily living. For people with blindness and low vision (BLV), environmental perception is compromised, creating myriad difficulties. Precise localization is still a gap in the field and is critical to safe navigation. Conventional GNSS positioning cannot provide satisfactory performance in urban canyons. 3D mapping-aided (3DMA) GNSS may serve as an urban GNSS solution, since the availability of 3D city models has widely increased. As a result, this study developed a real-time 3DMA GNSS-positioning system based on state-of-the-art 3DMA GNSS algorithms. Shadow matching was integrated with likelihood-based ranging 3DMA GNSS, generating positioning hypothesis candidates. To increase robustness, the 3DMA GNSS solution was then optimized with Doppler measurements using factor graph optimization (FGO) in a loosely-coupled fashion. This study also evaluated positioning performance using an advanced wearable system’s recorded data in New York City. The real-time forward-processed FGO can provide a root-mean-square error (RMSE) of about 21 m. The RMSE drops to 16 m when the data is post-processed with FGO in a combined direction. Overall results show that the proposed loosely-coupled 3DMA FGO algorithm can provide a better and more robust positioning performance for the multi-sensor integration approach used by this wearable for persons with BLV.

## 1. Introduction

Mobility and wayfinding are significant obstacles faced by people with BLV, specifically in urban areas. Degradation of the visual system can lead to a dramatic reduction in mobility. It has been shown that 80–90% of people with BLV spend the majority of their time inside buildings, and 30% rarely leave home alone [1,2].

Accurate positioning is essential for localization and navigation in urban canyons. Pedestrians with BLV who live in urban areas could benefit significantly from an integrated navigation solution for use during the activities of daily living. Much research has been performed to improve the autonomy of people with BLV, especially their ability to explore their environment. Rizzo et al. developed an advanced wearable in the form of an instrumented backpack equipped with microcomputers and sensors; this wearable incorporates cameras, inertial measurement units (IMUs), and GNSS positioning to provide a more comprehensive and full-featured navigation solution [3].

This study develops a real-time 3DMA GNSS-positioning system based on state-of-the-art 3DMA GNSS algorithms to advance the approach used for localization by the wearable. The integration of shadow matching and likelihood-based ranging 3DMA GNSS methods were selected to evaluate positioning hypothesis candidates’ likelihood scores. The position was then integrated with the velocity estimated by Doppler measurements using the FGO as loosely coupled.

Several experiments were designed in New York City (NYC) to acquire GNSS data during urban commuting (traveling between two defined locations, origin and target). A commercial-grade receiver, u-blox ZED-F9P, was connected to an Nvidia microcomputer, Jetson TX2. The performance of the proposed system was evaluated with the data recorded in a series of trips that took place on the lower east side of NYC (specifically Murray Hill, Manhattan). 

The remainder of this paper is organized as follows: Section 2 is an introduction to the integrated navigation system for pedestrians and existing studies. Section 3 is an introduction to the proposed positioning algorithm. Section 4 contains the designed experiment results and analysis. Finally, a conclusion and future work are presented in Section 5. 

## 2. Navigation System for Visually Impaired Pedestrians

### 2.1. Overview of Navigation System for Visually Impaired Pedestrians

The VIS^4^ION (Visually Impaired Smart Service System for Spatial Intelligence and Navigation), is an advanced wearable consisting of a backpack with wide-angle high-resolution cameras on the support straps; these cameras, with integrated microphones and IMUs, are connected to a light-weight, portable computer for real-time analysis [1,2,3,4,5,6,7,8]. This wearable system may be used by people with BLV during indoor and outdoor navigation, among other activities of daily living.

The platform provides real-time feedback using a binaural bone conduction headset and a haptic interface, allowing obstacle avoidance and situational awareness. More specifically, this mobile platform enables the users to understand their dynamically changing environment, giving them the agency to travel and wayfind independently. Our current **VIS^4^ION** system can process 720p video at 10 fps (dependent on the scene/task) and is robust without adding significant cognitive load to the end-user. This wearable runs off a laptop-battery with 66 Wh at 0.5 kg, yielding 3 h of run-time function.

### 2.2. Importance of GNSS Positioning

Precise and reliable positioning is required to support safe navigation services for persons with BLV. GNSS provides continuous positioning in the global frame in absolute coordinates. However, the performance of GNSS navigation in the urban environment is unsatisfactory. GNSS signals can be blocked or reflected over buildings, resulting in non-line-of-sight (NLOS) reception and the multipath effect [9]. These errors become more severe in highly urban cities with many high-rise buildings, such as Hong Kong and New York. As a result, researchers are trying to improve positioning by integrating different aids, such as inertial measurement units (IMU) and cameras.

One of the most frequently used approaches to integrating the GNSS with IMU is pedestrian dead reckoning (PDR) [10]. The integration of GNSS and IMU takes advantage of two approaches. GNSS positioning can provide absolute coordinates in a global frame. However, the availability is limited by the number of visible satellites. The IMU can provide continuous positioning without subjection to external factors. However, it can only offer relative incremental coordinates in the local frame. At the same time, IMUs suffer from a continuous bias that accumulates over time. As a result, GNSS/INS integration can provide a continuous positioning with absolute coordinates.

A camera is another popular aid for an integrated positioning system, which can provide the receiver’s orientation in the local frame. Visual odometry (VO) [11] can extract the features from the image and estimate the relative orientation change of the receiver. VO assumes that feature points are static. Matching two consecutive frames can aid positioning by providing relative position. However, VO is sensitive to illumination conditions and feature availability. In addition, because VO assumes that feature points are static, dynamic objects highly affect performance. When the feature points move in parallel with the camera, the system may think it remains static itself. Therefore, research indicates tightly coupled VO with INS improves positioning performance [12].

Research also suggests using a sky-pointing fisheye camera to detect the NLOS signal via image recognition algorithms [13,14]. Therefore, only the predicted healthy satellites are utilized for GNSS positioning. The limitation of excluding unhealthy satellites is that when many high-rise buildings surround the receiver, exclusion can result in bad satellite geometry and degrading performance.

Besides the aid of external sensors, improving the positioning accuracy of GNSS itself can definitely benefit the whole positioning system. Therefore, researchers are trying to identify and mitigate the NLOS error to improve the GNSS positioning alone. The consistency-check method [15] can detect and isolate unhealthy measurements and positioning performance can be improved to a satisfactory level. However, a consistency check will fail if the unhealthy measurements dominate the healthy ones [16].

Dual-frequencies measurements also demonstrate good performance in mitigating the multipath effects and isolating NLOS errors. Researchers used the nature of the higher resolution of L5-band measurements to design a new weighting scheme; the weighted least squares (WLS) method has been demonstrated to improve performance [17].

### 2.3. Related Works on 3DMA GNSS

One popular approach to improve GNSS positioning in urban canyons is using a 3D building model to identify and even correct the NLOS reception error. Different research has proposed to simulate the propagation path and model the error for code [18] and carrier phase [19] measurements. Research also incorporates the Fresnel zone analysis to predict GNSS multipath, NLOS, and diffraction effects in urban areas [20]. 3D building models demonstrate a massive improvement in positioning, namely 3D mapping-aided (3DMA) GNSS [21]. 3DMA GNSS has greatly impacted urban positioning in recent years, especially since huge improvements in smartphone positioning have been demonstrated [22]. An existing study uses the 3D building model to exclude the NLOS satellites; the weighted least squares (WLS) solution can therefore be enhanced [23]. However, we have to know the exact position to give a correct prediction and correction. As a result, the solution is usually determined as a particle-based approach. Position hypothesis candidates are distributed and measurements are modelled as the prediction at each candidate. The candidate with the highest similarity between modelled and actual received measurements is assumed to be the receiver location. Two basic categories of 3DMA GNSS algorithms are shadow matching and ranging-based 3DMA GNSS.

Shadow matching [24,25] matches the satellite visibility at different locations. The received satellites are assumed to be the LOS, while the non-received ones are assumed to be NLOS. The ephemeris provides the satellites’ position and matches the visibility at each candidate to find the highest visibility similarity. There is also research to further improve the shadow matching by particle filter [26].

Another 3DMA GNSS is the ranging-based method. The receiver location is determined by comparing the modelled and received pseudorange. Pseudorange measurements are modelled at each candidate. For the NLOS-predicted satellite, the NLOS reflection delay is also modelled based on a geometrical or statistical approach. The absolute position of the reflecting point has to be determined for the geometrical approach. A popular approach is ray-tracing [27,28]. It tests and validates the reflection path over each potential reflector, creating a high computational load. Therefore, research on using GPU to accelerate the computing process is relevant [29]. Moreover, an effective computational version called skymask 3DMA GNSS [30] was introduced. It determines the reflecting point over an enhanced skymask. Besides reflection delay, ray-tracing simulation can also calculate the GNSS signal strength based on the multipath propagation model [31,32]. A research study was conducted on the use of the ray-tracing technique to identify propagation obstructions and quantity propagation errors [33]. The study proposed measuring the position integrity as a set-based approach to bound the remaining systematic uncertainty. The statistical approach, also known as likelihood-based ranging [34], uses a skew–normal distribution to model the NLOS delay measurements and then remap the errors to the LOS with the normal distribution.

Performance of shadow matching and ranging-based 3DMA GNSS are different due to the healthy satellite and building geometry distribution. Shadow matching usually outperforms in the across-street direction, while ranging-based 3DMA GNSS obtains higher accuracy in the along-street direction. The complementary nature of the two approaches inspired researchers to integrate them. The latest study shows that an integrated solution of 3DMA GNSS can provide positioning accuracy of around 10 m or less in urban canyons with both single-frequency [34] and multi-frequency [35] measurements.

However, most of these approaches only concern positioning in a single epoch. Hence the performance is not robust for continuous positioning. As a result, a temporal connection is required to improve the reliability of urban GNSS positioning. There is research using the Kalman filter (KF) and extended Kalman filter (EKF) to recursively update the recent state through the prediction based on past estimation and error of current measurements [36,37]. Researchers also use particle filters to effectively distribute and sample the candidates [26,38]. Moreover, a grid filter was adopted to distribute positioning candidates evenly [34]. The filtering techniques demonstrate excellent results in improving the smoothness of the positioning solution. Meanwhile, a machine learning approach can intelligently predict the change of signal status and estimate the most likely path on the map as the optimized positioning estimation [39].

Another approach is using factor graph optimization (FGO) [40], which optimizes the states of all epochs with many constraints. The solution will be highly robust as FGO optimizes all temporal constraints as a batch approach. Researchers open-sourced the FGO code that integrates GNSS positioning with Doppler measurements to provide a multi-epoch optimized solution [41]. FGO also applies to centimeter-level accuracy positioning via carrier-phase measurements, such as GNSS PPP [42] and RTK [41]. Furthermore, 3DMA GNSS-based collaborative positioning can benefit from using FGO [43] to optimize the performance of multi-agent collaborative positioning.

Different research demonstrates that FGO can provide an excellent positioning performance. This study integrates 3DMA GNSS with velocity estimated by Doppler measurements as a loosely-coupled solution, and states were optimized via FGO. The integrated solution can provide a more robust trajectory for pedestrian applications, such as this wearable.

## 3. Proposed Real-Time 3D Mapping-Aided (3DMA) GNSS-Positioning System

**Figure 1:**
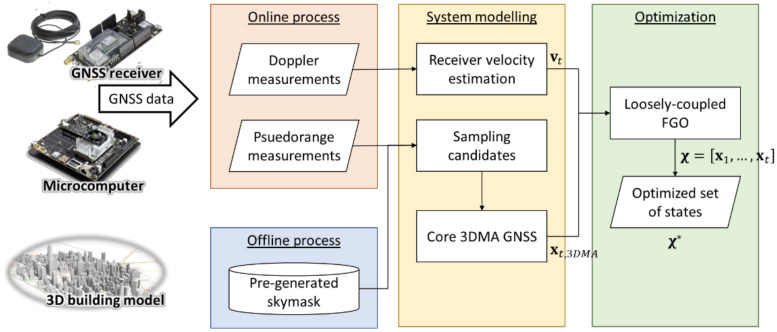
System flowchart on the proposed system.

This section introduces the proposed loosely-coupled 3DMA GNSS- and velocity-positioning system via FGO. The flowchart is shown in Figure 1.

### 3.1. Open-Sourced 3D City Models

**Figure 2:**
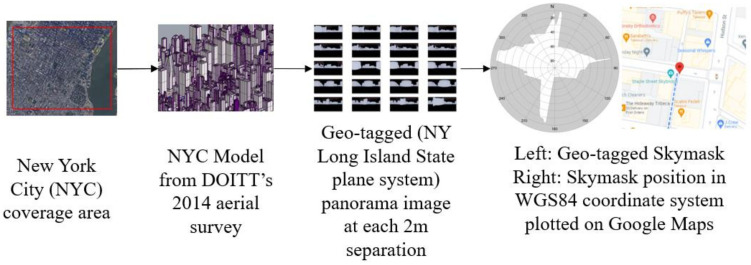
Geo-tagged skymask generation from NYC 3D model.

3D models of New York City (NYC) were obtained from open-sourced repositories released by the Department of Information Technology & Telecommunications’ (DoITT) 2014 aerial survey [44] with a level of detail (LoD) of 2. The city model was registered to cartesian New York Long Island State Plane FIPS 3104 coordinates. The city model is shown in Figure 2.

### 3.2. Offline Stage Skymasks Generation

A skymask is a skyplot with building boundaries for a single location. It is an array with a total of 360 entries that represents the azimuth angle from 0° to 359°. Each entry stores the highest elevation angle of the building blockage in degrees in the corresponding entry (each azimuth angle). Skymasks are generated in an offline stage. The intentional coverage area for the 3DMA GNSS was first selected, and then the 3D city models were downloaded. The models were imported into the Rhino 7 3D-modelling software [45], and then converted to the Unreal engine-supported format for automated skymask generation [46]. The selected area was separated into 4 m catchment areas for each potential location to capture a 360° equirectangular image of the building outline. The skymask generation process was then performed by setting up a virtual camera in the Unreal engine to capture the panorama image at each potential location outside the building and above the terrain. The camera height was set manually based on the covered area, which was 15 m in this study, to best accommodate the elevation variation across the testing area. The elevation of the potential location can be set based on the digital terrain model for mass generation. Saved panorama images were then classified into obstacles and sky. At the cutting edge between obstacles and the sky, pixels were converted to angular position (azimuth and elevation angle) at the skymask. Lastly, each skymask corresponded to one position in the state plane coordinate system, which was converted to WGS84 for real-time 3DMA GNSS positioning. The extracted skymask at each available location was then saved to a specified format for the microcomputer to use during real-time positioning [30].

### 3.3. 3DMA GNSS Positioning Algorithm

3DMA GNSS evenly distributes the hypothesis positioning candidates during the online stage around the initial position. After that, the simulated measurements are generated to be compared with the received measurements for each candidate. Due to their computational efficiency, this study integrates shadow matching and likelihood-based ranging 3DMA GNSS. The implementation can be found in [35].

#### 3.3.1. Skymask Context-Based Candidates Sampling

An effective hypothesis positioning candidates sampling is important for 3DMA GNSS. The sampling area must cover the receiver location to achieve the theoretically best performance. Enlarging the sampling radius ensures the receiver location is being covered. However, this creates a massive computational load for the low-end microcomputer, which is not practical for a real-time application. Required computational time is proportional to the number of sampled candidates and received satellites. The computational time is within the necessary output rate. Thus, we proposed to use the surrounding skymasks and principal component analysis (PCA) to determine the street direction and distribute the sampling candidates effectively.

Candidate distribution is based on the weighted least squares (WLS) for the first epoch and using the previous epoch FGO solution as the initial position after it is available. An initial circle with a sampling radius, R, e.g., 50 m, is set up empirically to estimate the surrounding environment by weighted averaging skymask, ${\bar{SM}}^{az}$.

(1) $${\bar{SM}}^{az}=\frac{1}{\sum w_{k}}\sum_{k=1}^{K}w_{k}SM_{P_{k}}^{az}\;\mathrm{where}\;P_{k}\in \left\{\left|P-x_{init}\right|<R\right\}$$

where $SM_{P_{k}}^{az}$ is the skymask of location $P_{k}$ where it is within the sampling radius R based on the initial location, $x_{init}$. az is the array index that represents the azimuth angle from 0° to 359°. $w_{k}={\left|P_{k}-x_{init}\right|}^{2}$ is the weighting of location $P_{k}$ based on the distance between the initial location, $x_{init}$.

The averaged skymask is then converted to vectors in the Earth-Centered-Earth-Fixed (ECEF) frame together with the transformation matrix, R, expressed as,

(2) $$q^{az}=R\cdot \left[\sin az\cdot \cos{\bar{SM}}^{az},\cos az\cos{\bar{SM}}^{az}\right]$$

where R is the transformation matrix that converts the vector in the local frame to the world frame in ECEF. Thus, we can form the variance–covariance matrix, Q,

(3) $$Q=q^{T}q$$

Therefore, we can obtain the eigenvalues, $\lambda =\left[\begin{array}{cc} \lambda_{1} & 0\\ 0 & \lambda_{2} \end{array}\right]$, and eigenvectors, $V=\left[\begin{array}{cc} v_{1} & v_{2} \end{array}\right]$, from the variance–covariance matrix, Q. Note that the eigenvalues and eigenvectors are sorted in descending order, e.g., $\lambda_{1}$ and $v_{1}$ denotes that they are with the largest eigenvalue. In addition, the eigenvector with a larger eigenvalue can be interpreted as the street’s longitudinal direction.

Finally, we can filter the initial circle with the ellipsoid based on the determined eigenvalues and eigenvectors. The length of the semi-major and semi-minor axes are R and R·λ2λ1, respectively. The direction of the semi-major and semi-minor axes are $v_{1}$ and $v_{2}$, respectively.

(4) pj=1…J=Pk∈dk·v12R2+dk·v22R·λ2λ12<1

where $d_{k}=P_{k}-x_{init}$ is the vector between the candidate’s position, $P_{k}$, and initial location, $x_{init}$.

The distributed candidates are an ellipsoid with a semi-major axis of 50 m. The separation for each candidate is 4 m. The above settings were determined empirically and suitable for real-time processing on the low-end microcomputer used in this study. A semi-major axis of 50 m can cover the position error of the initial position in most cases. In comparison, separation with 4 m can reduce the number of distributed candidates while maintaining an acceptable accuracy level.

**Figure 3:**
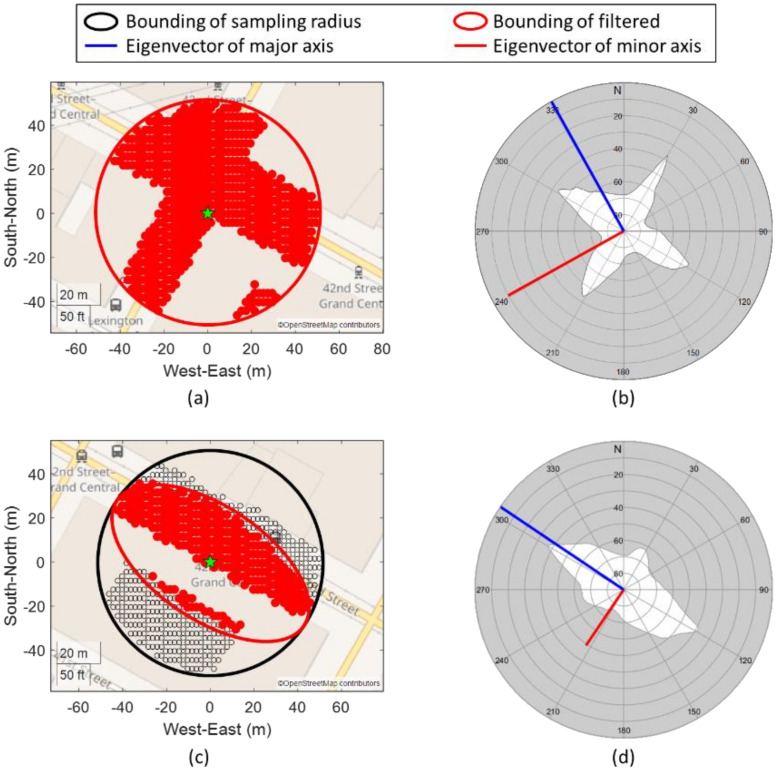
Typical cases of candidate sampling in urban environments on intersection (**a**,**b**) and straight street (**c**,**d**). Note that the eigenvectors (red and blue lines in (**b**,**d**) are projected back to azimuth and elevation angle (local frame) for illustration here.

The proposed distribution can effectively distribute the hypothesis position candidates based on the surrounding environment. Figure 3 shows two typical cases in urban canyons. In road intersections, two eigenvalues are nearly the same (Figure 3b), such that the candidates’ distribution is almost a circle that covers the whole intersection, as shown in Figure 3a. In contrast, when the initial location is in a straight street, the largest eigenvalue is much larger than the other (Figure 3d). The candidates are most likely distributed on the same street but not the next block.

**Figure 4:**
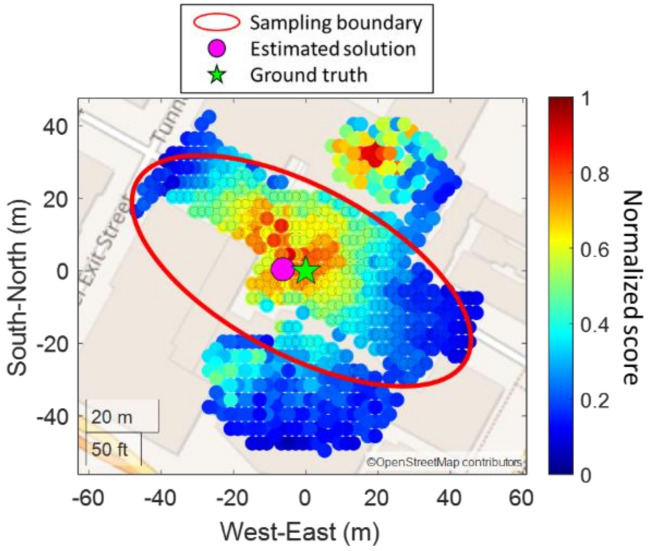
Example of skymask context−based candidates sampling.

The prevention of candidate distribution at the next block can potentially mitigate the local minima issue caused by the high similarity of building geometry, as shown in Figure 4. Local minima are located on the next street (the high score part in red near the upper right corner). After applying the proposed skymask context-based candidates sampling strategy, the local minima issue can be mitigated.

To conclude, the skymask context-based candidates sampling can effectively distribute the position candidates. It has two main advantages. The first is to reduce the computational load by reducing the number of distributed candidates based on the surrounding environment. The second advantage is that candidates are most likely distributed on the same street. Therefore, the local minima on the next street can potentially be excluded.

#### 3.3.2. Integrated Solution of 3DMA GNSS

For each candidate $p_{j=1\ldots J}$, the integrated likelihood score, $S_{j,SDM+LBR}$, will be evaluated,

(5) $$S_{j,SDM+LBR}=\sqrt{S_{j,\mathrm{LBR}}\times S_{j,\mathrm{SDM}}}$$

where $S_{j,\mathrm{LBR}}$ and $S_{j,\mathrm{SDM}}$ are the likelihood score of likelihood-based ranging 3DMA GNSS and shadow matching, respectively. The detail of the calculation of the likelihood scores can be found at [35].

Shadow matching evaluates the visibility consistency between the measured carrier-to-noise ratio ($C/N_{0}$) and prediction with skymask. Shadow matching requires all satellites in ephemerides to predict the non-received one. Implementation-wise, we automatically download ephemerides from the day prior. Additionally, we use the same receiving time but a day before to estimate the satellite’s angular position for visibility prediction with skymask. If the internet is enabled for the execution platform this can be replaced by assisted GNSS (AGNSS) [47,48] and provide the satellite data to determine position via standard protocol, such as secure user plane location (SUPL). This is more easily achieved with built-in AGNSS devices, such as smartphones [49].

With likelihood-based ranging 3DMA GNSS, we model the pseudorange at each candidate position and compare it with the received pseudorange measurements. The NLOS predicted satellite at a candidate, likelihood-based ranging 3DMA GNSS remaps NLOS pseudorange difference to a LOS one using the distribution model.

The integrated solution of 3DMA GNSS, $x_{3\mathrm{DMA}}$, is calculated by weighted averaging of the distributed candidates with their likelihood score,

(6) $$x_{3\mathrm{DMA}}=\frac{\sum_{j=1}^{J}p_{j}S_{j,SDM+LBR}}{\sum_{j=1}^{J}S_{j,SDM+LBR}}$$

The receiver location is then optimized via FGO as a loosely-coupled solution.

### 3.4. Loosely-Coupled Factor Graph Optimization (LC-FGO)

**Figure 5:**
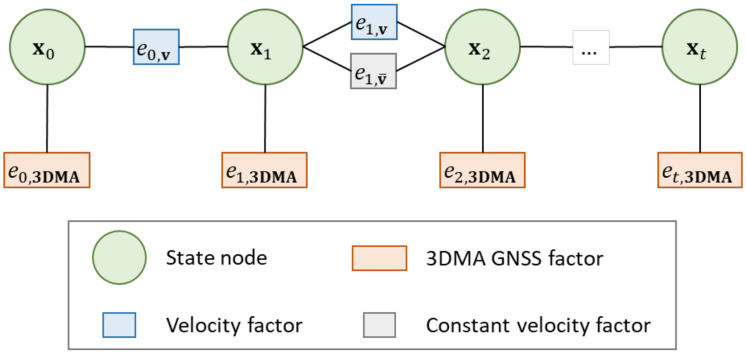
Structure of the proposed loosely-coupled 3DMA GNSS and velocity via FGO.

This study also optimized the 3DMA GNSS solution as a batch via forming the graphical optimization. It is associated with FGO, connecting two consecutive epochs’ solutions with velocity. The overall structure of the FGO process is shown in Figure 5.

The error factor between the 3DMA GNSS solution, $x_{t,3DMA}$, and optimized state, $x_{t}$, is given by,

(7) $$\|e_{t,3\mathrm{DMA}}{\|}_{\sigma_{3\mathrm{DMA}}^{2}}^{2}=\|x_{t}-x_{t,3\mathrm{DMA}}{\|}_{\sigma_{3\mathrm{DMA}}^{2}}^{2}$$

where $\sigma_{3\mathrm{DMA}}^{2}=\alpha \cdot \mathrm{diag}\left(\left[\sigma_{3\mathrm{DMA},x}^{2},\sigma_{3\mathrm{DMA},y}^{2},\sigma_{3\mathrm{DMA},z}^{2}\right]\right)$ is a diagonal variance matrix of the 3DMA GNSS. Constant α=1 is an empirically determined tuning factor for 3DMA GNSS error factor. Variance at each axis is taken by the distance variation between the 3DMA GNSS solution and candidates with the highest 10% likelihood score, divided by the separation of candidates, γ,

(8) $$\sigma_{3\mathrm{DMA}}^{2}=\frac{1}{\gamma }\mathrm{Var}\left(\left|x_{t,3DMA}-x_{t,10\%}\right|\right)$$

where $x_{t,10\%}$ represents the candidates’ position with the highest 10% likelihood score. $\left|\cdot \right|$ denotes the Euclidean distance between two positions.

Receiver velocity, $v_{t}$, and clock drift, $c{\dot{\delta t}}_{t}$, is estimated by the Doppler measurements of every satellite i at epoch t, $d_{t}=\left[d_{t}^{1},\ldots ,d_{t}^{i}\right]$, via the least-squares (LS) method [41]. The error factor between consecutive epochs can be expressed as follows,

(9) $$\|e_{t,v}{\|}_{\sigma_{v,t}^{2}}^{2}=\|v_{t}-\frac{1}{\Delta t}\left(x_{t+1}-x_{t}\right){\|}_{\sigma_{v,t}^{2}}^{2}$$

where Δt is the time difference between epoch t and t+1. $\sigma_{v}^{2}=\beta \cdot \mathrm{diag}\left(\left[\sigma_{v,x}^{2},\sigma_{v,y}^{2},\sigma_{v,z}^{2}\right]\right)$ is a diagonal covariance matrix associated with the velocity $v_{t}$ at *x*-, *y*-, and *z*-axis, respectively. And constant β=5.2 is an empirically determined tuning factor for velocity error factor. Parameters α and β are determined empirically based on an open-source dataset [50] that covers different typical urban canyon scenarios. All results in this study share the same set of parameters. If tuning factor β increases, the integrated result approaches 3DMA GNSS more. If decreasing the factor below 5.0, the optimized results will be much smoother, but easier to observe a drift if a wrong velocity is estimated.

A constant velocity motion model [51] is included in this graph structure to provide a smoothed trajectory estimation. As this study assumed, users’ motions are small with an ignorable acceleration. This factor minimizes the error between the position change between two epochs and the averaged velocity estimated via Doppler measurements, modelled as follows,

(10) $$\|e_{t,\bar{v}}{\|}_{\sigma_{\bar{v}}^{2}}^{2}=\|\frac{1}{2}\left(v_{t}+v_{t+1}\right)-\frac{1}{\Delta t}\left(x_{t+1}-x_{t}\right){\|}_{\sigma_{\bar{v}}^{2}}^{2}$$

where $\sigma_{\bar{v}}^{2}=\frac{1}{2}\left(\sigma_{v,t}^{2}+\sigma_{v,t+1}^{2}\right)$ is the averaged diagonal covariance matrix at time t and t+1.

The cost function for the position estimation of the proposed loosely-coupled 3DMA GNSS via FGO is formulated as,

(11) $$\chi^{*}=\underset{\chi }{\mathrm{argmin}}\sum_{t}\|e_{t,3\mathrm{DMA}}{\|}_{\sigma_{3\mathrm{DMA}}^{2}}^{2}+\|e_{t,v}{\|}_{\sigma_{v,t}^{2}}^{2}+\|e_{t,\bar{v}}{\|}_{\sigma_{\bar{v}}^{2}}^{2}$$

where $\chi =\left[x_{1},\ldots ,x_{t}\right]$ is the state set of the receiver and $\chi^{*}$ denotes the optimal states set. For computational efficiency, a sliding window for FGO is set as 200 s, which is determined empirically.

## 4. Experiments and Results

### 4.1. Experiment Setup

A commercial-grade receiver, u-blox ZED-F9P, was connected to a microcomputer, Nvidia Jetson TX2. A total of four satellite constellations with a single frequency were enabled during the experiments: GPS L1, GLONASS G1, Galileo E1, and Beidou B1. We modified the open-source library RTKLIB [52] for the GNSS-related processes, the main program structure can be found in Appendix B. Google Ceres Solver [53] was used for the nonlinear least squares (NLS) and FGO processes. Several experiments took place on the lower east side of NYC (Murray Hill, Manhattan), map plot can be found in Appendix A. In these experiments, two team members walked fixed navigation routes as if commuting between an origin (NYU Medical Center, New York City) and specific target destinations (storefronts) in a 1 mile radius. A total of 11 trips were made and used for analysis.

The ground-truth reference trajectory was obtained via post-processing. The pedestrian subjects who collected the data walked straight lines and made their best attempts not to veer. Starting and ending locations and locations in-between were labelled manually. We also equipped a smartphone during the experiment and recorded the device location output. We used the smartphone output location to interpolate the longitudinal speed and project the vector between starting and ending location.

### 4.2. Experiment Results

The evaluation was aimed at comparing the proposed algorithms in both a real-time and post-processing manner, also with several conventional solutions:

1. **NMEA**: receiver output solution.
2. **WLS**: weighted least squares method [52]; uses pseudorange to estimate receiver location.
3. **3DMA GNSS**: snapshot state-of-the-art 3DMA GNSS with positioning hypothesis candidates [35].
4. **LC-FGO (proposed)**: real-time forward (instantaneous) processed loosely-coupled FGO solution with integrated 3DMA GNSS and velocity.
5. **LC-FGO-PP (proposed)**: combined (forward and backward) processed loosely-coupled FGO solution with integrated 3DMA GNSS and velocity.

The optimization frame was under the ECEF coordinate system. The comparison is divided into root-mean-squared error (RMSE) and standard deviation (STD) positioning error in meters. Note that both LC-FGO and LC-FGO-PP share the same graph structure. Only LC-FGO-PP uses historical and future factors and is optimized in a combined direction forward and backwards.

A total of 11 experimental navigation trips were conducted in New York City. The positioning results of different trips are shown in Table 1. In summary, the candidate-based 3DMA GNSS always outperformed the conventional WLS. After integrating the velocity and optimizing it only for forward direction, the positioning accuracy was improved. If constraints optimization is performed in a combined manner, the positioning accuracy becomes higher. Meanwhile, in most cases, FGO outperformed the receiver’s output solution (NMEA). From the overall performance of different experiments, the RMSE and STD of 3DMA GNSS are 25.34 m and 19.46 m, respectively. At the same time, LC-FGO is 21.05 m and 14.60 m for RMSE and STD, respectively. LC-FGO-PP have a stronger constraint between epochs and obtained RMSE of 15.97 m while STD is 12.48 m. Two FGO have a smaller RMSE, which means that the overall performance is better than that of 3DMA GNSS, and a lower STD implies that they are more robust. We selected two trips out of eleven (one good and bad case, respectively) to further discuss in this section.

**Figure 6:**
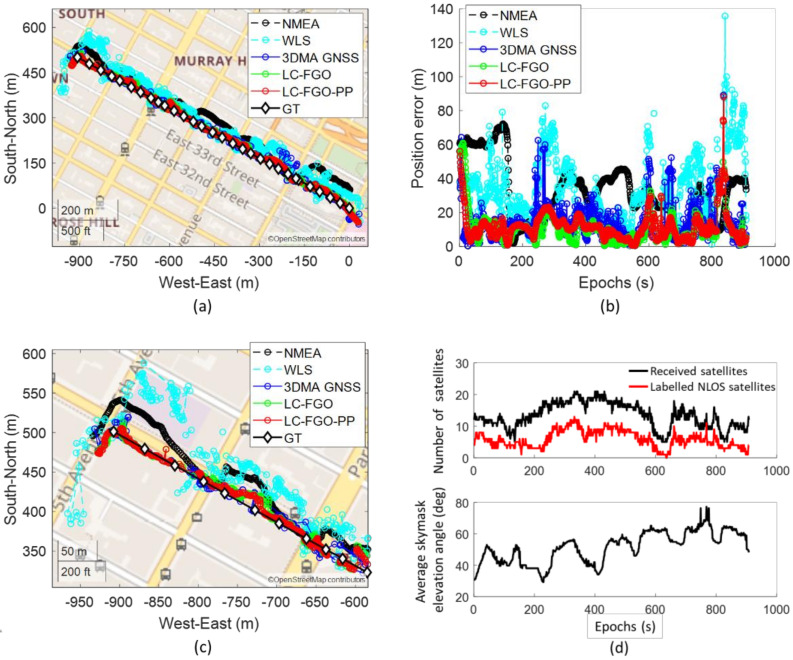
(**a**) map plot, (**b**) positioning errors, (**c**) magnified map plot of last 300 epochs, and (**d**) number of received satellites and average skymask elevation angle of good positioning trip (Trip 6).

We first present a trip with a good positioning performance (Trip 6). It starts from a relatively open-sky area and travels along straight to a deep urban canyon. The plots are shown in Figure 6.

In this experiment, it can be observed that the 3DMA GNSS outperforms WLS by twofold, and the positioning RMSE are 18.27 m and 39.17 m, respectively. Many solutions for WLS were located on the opposite side or on the wrong street, as shown in Figure 6c. With the aid of 3D models, 3DMA can correct the solution back to the correct street. If further integrated with the Doppler measurements, the positioning error can be suppressed in most cases, especially around epochs 200 s to 600 s. Results in the RMSE of the forward LC-FGO and combined FGO (LC-FGO-PP) are 15.32 m and 14.56 m, respectively.

**Figure 7:**
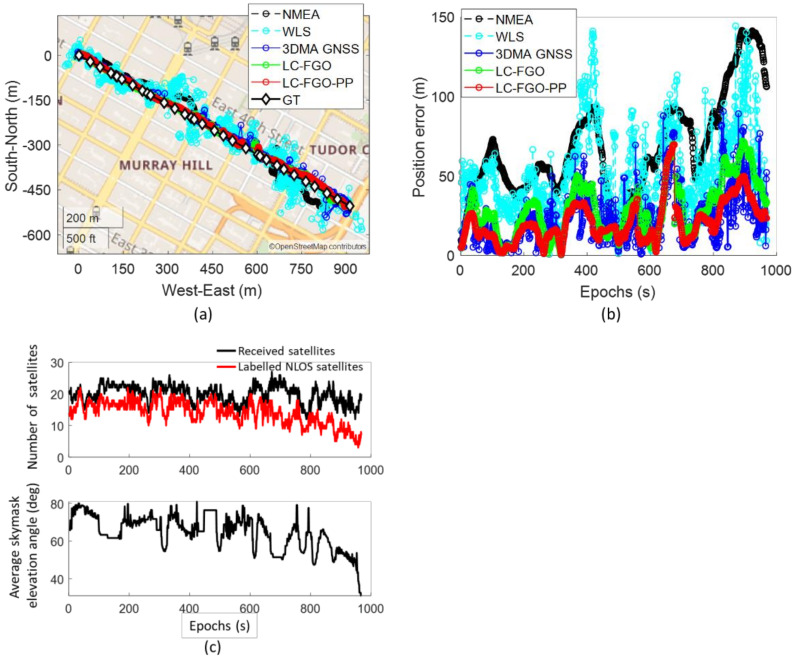
(**a**) map plot, (**b**) positioning errors, and (**c**) number of received satellites and average skymask elevation angle of bad positioning trip (Trip 2).

Trip 6 is followed by a navigation trip with bad positioning results (Trip 2). Trip 2 begins in a deep urban canyon with a walk along the street to a relatively open area which is the opposite to Trip 6. The map and error plot of this experiment are shown in Figure 7.

Similar to Trip 6, both 3DMA GNSS and two LC-FGO algorithms outperform WLS. The RMSE of WLS, 3DMA GNSS, LC-FGO, and LC-FGO-PP are 59.15 m, 29.14 m, 33.56 m, and 24.66 m, respectively. However, the LC-FGO is not outperforming the 3DMA GNSS. The overall positioning error is larger than that in Trip 6 because the environment is more complex. The average skymask elevation angle is higher in Trip 2, resulting in a more severe NLOS reception that mostly occupies a large portion of the total received satellites. The main error comes from the last 200 epochs. The performance of 3DMA GNSS keeps fluctuating during this period. The natural difference between 3DMA GNSS and FGO results in the average performance of this experiment. 3DMA GNSS is a snapshot estimation, and each epoch performance is independent of the others. However, the FGO is different, especially for the forward FGO. The fluctuation of the forward FGO will keep accumulating errors in batch optimization. Therefore, the future estimation is affected. However, the combined FGO, LC-FGO-PP, has a much stronger constraint that tries to optimize the solution in both directions. As a result, the positioning error can be suppressed. Therefore, if the performance of LC-FGO has to be improved, marginalization analysis must be done to find the acceptable error of this graphical problem. And we must adaptively select the existing trustworthy information in the sliding window.

**Figure 8:**
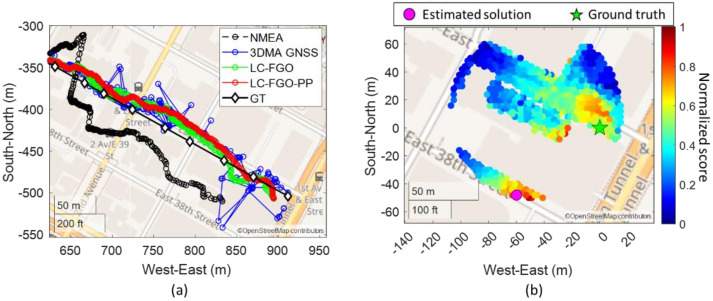
(**a**) zoom−in map plot near the end of Trip 2. (**b**) one of the epochs with large position error due to local minima problem.

Near the end of the experiment, some 3DMA GNSS solutions wrongly estimated the position of the next block, as shown in Figure 8a. The reason is that the receiver was located in a relatively open area. The PCA result of the average skymask indicates that the two eigenvalues are similar, and there are no clear major or minor axes. Results in the candidate distribute as a full circle, and the solution estimates at the local minima, as shown in Figure 8b. The candidate might have to distribute based on the user’s average historical motion to resolve this issue. However, a pedestrian’s motion is not as consistent as a vehicle’s, therefore distributing candidates based on average motion cannot capture a rapid motion change. Another consideration is the detection of an instant motion change with an inertial measurement unit (IMU) that could be integrated into the camera or platform, more broadly.

**Figure 9:**
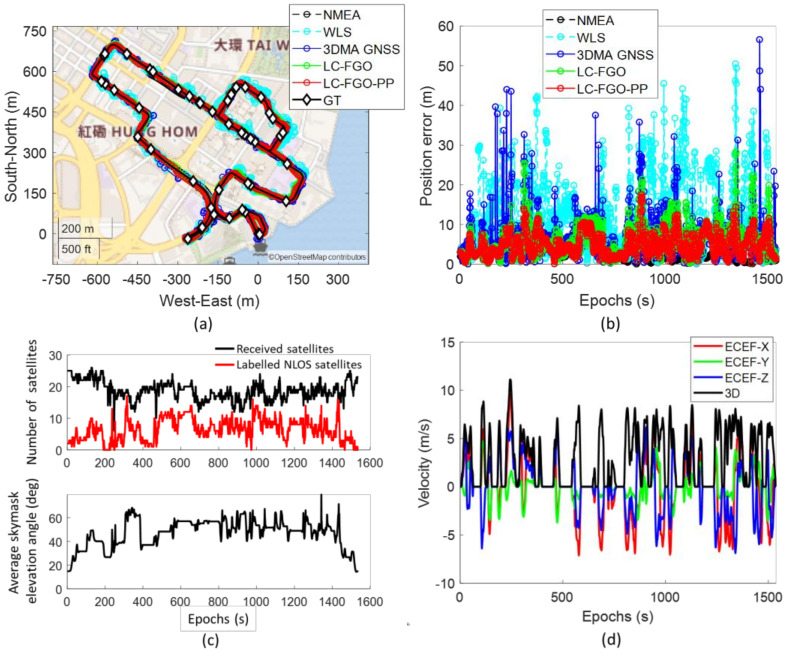
(**a**) map plot, (**b**) positioning errors, (**c**) number of received satellites and average skymask elevation angle, and (**d**) velocities under the ECEF coordinate system provided by NovAtel SPAN−CPT of the vehicle-mounted experiment.

Lastly, we also demonstrate a vehicle case in Hong Kong using the same receiver. The experiment covers different scenarios of an urban city, from open-sky areas to deep urban canyons. The data can be found in [50]. The data is collected using the same receiver model, u-blox ZED-F9P, with a patch antenna. The reference trajectory is provided by NovAtel SPAN-CPT [54], a GNSS RTK/INS (fiber-optic gyroscopes, FOG) integrated navigation system. Positioning statistics are shown in Table 2, and the map plot and error plot are shown in Figure 9. The vehicle case covers more scenarios across different environmental complexities and velocities, as shown in Figure 9c,d, respectively. Urban scenarios covering an average skymask elevation angle of around 20 degrees up to nearly 80 degrees are covered. And velocities from 0 m/s up to about 12 m/s are presented in this case.

**Figure 10:**
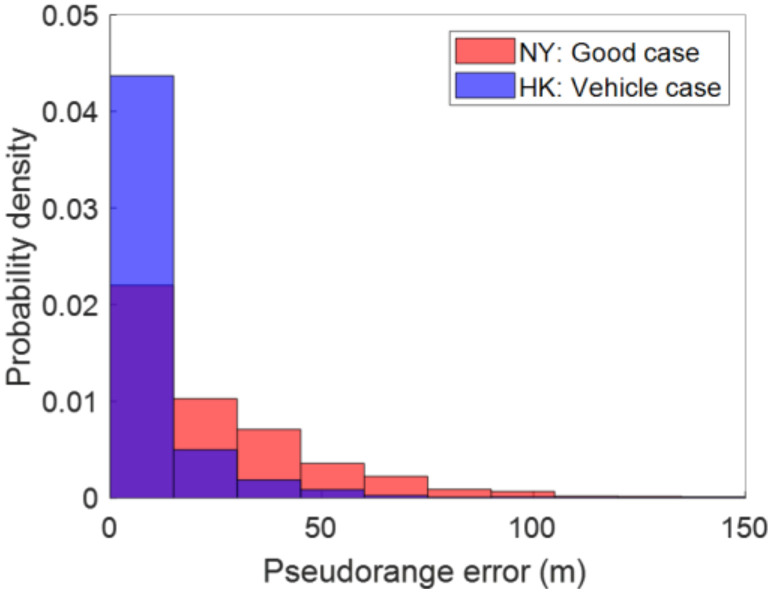
Probability density function plot on pseudorange error labelled by double differencing technique. Note that the master satellite is excluded from pseudorange error labelling, e.g., i≠m in Equation (12).

Both 3DMA GNSS and FGO outperformed WLS in this case. RMSE of WLS, 3DMA GNSS, LC-FGO, and LC-FGO-PP are 14.92 m, 7.94 m, 8.09 m, and 5.80 m, respectively. Overall, the positioning outperformed what was noted for New York. There are two main reasons for the excellent positioning performance. Firstly, the local environments in NY and HK were different; the testing areas in NY were more urbanized, i.e., the average skymasks’ elevation angle at ground truths in all navigation trips was 58.6° and 46.9° for New York and Hong Kong, respectively. Secondly, measurement noise was notably higher in NY, likely secondary to motion variation [55]. The vehicle (HK) had higher dynamic motion, and measurements suffered less from the multipath effects, therefore better positioning performance could be obtained. We labelled the pseudorange error using the double differencing technique [56] for a good case in the New York dataset (Trip 6) and Hong Kong, as shown in Figure 10. The double difference [56] requires measurements from the reference station. The pseudorange of the commonly received satellites is differenced. The common clock and atmospheric errors are eliminated. Geometric distance, D, is given by the calculated satellite position from the ephemeris, surveyed location of the reference station, and our labelled ground truth. The reference station was set up in an open-sky area where measurement can be assumed to be healthy. Therefore, the remaining value after differencing can be treated as the error caused by the environment of our receiver location. The double-difference-labelled pseudorange error of the i-th satellite, $\nabla \Delta \rho^{i}$, can be calculated by,

(12) $$\begin{array}{c} \nabla \Delta \rho^{i}=\rho_{rcv}^{i}-\rho_{rcv}^{m}-\left(\rho_{ref}^{i}-\rho_{ref}^{m}\right)-\nabla \Delta D^{i}\\ \mathrm{where}\;\nabla \Delta D^{i}=D_{rcv}^{i}-D_{rcv}^{m}-\left(D_{ref}^{i}-D_{ref}^{m}\right) \end{array}$$

where ${*}_{rcv}$ stands for receiver data while ${*}_{ref}$ stands for reference station data. ${*}^{m}$ stands for the master satellite’s data. It is selected in a system-specific pivot satellite manner with the highest elevation angle. ρ and D stand for pseudorange measurement and geometric distance, respectively. Reference station data was retrieved from NYS Spatial Reference Network (NYSNet) for data evaluation in New York. For Hong Kong dataset evaluation, reference station data was retrieved from Hong Kong Satellite Positioning Reference Station Network (SatRef).

The HK dataset reveals a better pseudorange quality, and it is reasonable to expect better positioning performance.

**Figure 11:**
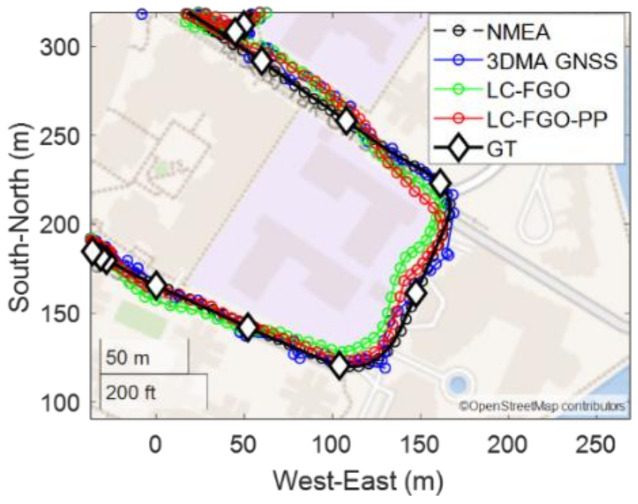
Zoom-in map plot of positioning error caused by badly estimated velocity.

A similar conclusion can be made in this vehicle-mounted experiment based on the results. 3DMA GNSS and LC-FGO obtain similar performance in this data, but comparing the positioning error shown in Figure 9b, LC-FGO can reduce the positioning better than the 3DMA, resulting in a smaller standard deviation on the positioning error. In other words, LC-FGO can provide a smoother and more robust trajectory, which applies to LC-FGO-PP. However, the error of velocity can degrade the integration performance. Figure 11 shows the epoch around 1300 s. Although the 3DMA GNSS performs well, the wrong velocity estimated by Doppler measurements with WLS results in wrong integrated results. As a result, error mitigation or a correction for Doppler measurements have to be explored in the future. Therefore, tightly coupling these approaches with Doppler measurements can potentially address the problem. In doing so, wrong Doppler measurements will be identified and isolated from the state estimation separately. A sophisticated model may be developed to model the Doppler errors [57] so that inaccurate measurements are used in the future. Meanwhile, 3DMA GNSS can be integrated with Doppler measurements more tightly in future work by expressing discrete sampled locations with a continuous mathematical model.

### 4.3. Computational Load and Storage Requirements

One of the main contributions of this study is to develop a real-time positioning system. Therefore, the processing time of a single epoch solution is important to guidance for a real-time operation that needs to maintain an output rate of 1 Hz. The computational load is directly proportional to the number of distributed candidates (sampling radius) and available satellites. From the result, the average number of total received satellites (including LOS and NLOS) and sampled candidates are 26 and 1143, respectively. The processing time for a single epoch solution is 0.91 s. In other words, the implemented system can provide a real-time operation at a 1 Hz output rate. If a higher output rate is required, using GPU has a huge potential to accelerate the process for real-time applications, such as presented work on using GPU for ray-tracing simulation [29] and correlation-level positioning [58].

Another important point for 3DMA GNSS implementation is the format employed to store the information of 3D building models. It is impossible for a microcomputer to generate skymask online or in real-time. Therefore, the skymask is pre-generated offline and stored in CSV format, as in [50]. If we were to cover the New York downtown area (around 3.6 km by 2.9 km), a total of 812,403 locations (outside the buildings) with 4 m separations for each candidate, the total file size of requisite skymasks would be 1.30 GB. This storage is still manageable for city-scoped applications. If the system has to be extended state-wise, further engineering work must be done to devise a sustainable solution for skymask database implementation.

## 5. Conclusions and Future Work

This study developed a real-time loosely-coupled 3DMA GNSS with a Doppler measurements positioning system via FGO, and skymask context-based candidate sampling. Our approach distributes the candidates more effectively and mitigates local minima issues. Based on the experimental results, the positioning RMSE of loosely-coupled 3DMA GNSS with Doppler measurements via FGO is around 21 m with STD 15 m (on average). Performance can be further improved when optimizing in a combined direction with RMSE reduced to about 16 m with a STD of 13 m. The FGO can provide a lower standard deviation error than the candidate-based 3DMA GNSS, which means that it can provide a smoother and more robust solution.

However, the performance of LC-FGO still has space to be improved. The results show that candidate-based 3DMA GNSS intermittently outperforms LC-FGO. The reason is the positioning error of 3DMA GNSS keeps contributing to the integration with Doppler measurements. Accumulated error results affect future batch optimization. An adaptive scheme should be developed to select the high confidence information in the sliding window. Moreover, tighter integration of the 3DMA GNSS with Doppler measurements should be effected to improve the performance.

Furthermore, bad Doppler measurements result in wrongly estimated velocity. This will degrade the FGO performance. Doppler measurements error mitigation or correction is the key to improving the positioning. In future research, we will explore how 3DMA GNSS can more tightly integrate with Doppler measurements to provide a more robust positioning in the urban canyons for smart health applications and beyond.

## Figures and Tables

**Table 1:** Statistics on positioning results of all experiments.

Navigation Trips	Epochs (s)	Algorithm	RMSE (m)	STD (m)
1	952	1. NMEA	31.09	14.47
		2. WLS	38.30	20.00
		2. 3DMA GNSS	19.70	15.51
		3. LC-FGO	24.66	14.95
		4. LC-FGO-PP	15.54	12.25
2	979	1. NMEA	74.81	31.57
		2. WLS	59.15	26.94
		2. 3DMA GNSS	29.14	16.75
		3. LC-FGO	33.56	17.13
		4. LC-FGO-PP	24.66	13.08
3	574	1. NMEA	19.87	7.35
		2. WLS	62.66	38.62
		2. 3DMA GNSS	27.62	16.40
		3. LC-FGO	22.98	11.04
		4. LC-FGO-PP	21.38	9.51
4	607	1. NMEA	17.20	11.08
		2. WLS	91.98	54.99
		2. 3DMA GNSS	21.08	12.26
		3. LC-FGO	13.01	6.48
		4. LC-FGO-PP	14.09	6.85
5	599	1. NMEA	29.01	7.43
		2. WLS	30.34	10.46
		2. 3DMA GNSS	22.64	13.21
		3. LC-FGO	20.38	10.25
		4. LC-FGO-PP	18.90	11.18
6	934	1. NMEA	36.89	18.01
		2. WLS	39.17	19.54
		2. 3DMA GNSS	18.27	11.28
		3. LC-FGO	15.32	10.44
		4. LC-FGO-PP	14.56	8.73
7	885	1. NMEA	33.36	15.61
		2. WLS	44.25	25.89
		2. 3DMA GNSS	18.64	11.27
		3. LC-FGO	25.17	11.03
		4. LC-FGO-PP	12.17	6.08
8	513	1. NMEA	39.09	11.05
		2. WLS	36.43	15.94
		2. 3DMA GNSS	16.55	9.46
		3. LC-FGO	21.30	8.36
		4. LC-FGO-PP	14.22	7.47
9	878	1. NMEA	24.17	10.38
		2. WLS	40.86	21.21
		2. 3DMA GNSS	41.50	26.91
		3. LC-FGO	44.62	29.99
		4. LC-FGO-PP	37.67	24.91
10	742	1. NMEA	36.01	16.88
		2. WLS	49.43	31.11
		2. 3DMA GNSS	26.72	15.29
		3. LC-FGO	25.49	13.08
		4. LC-FGO-PP	20.46	11.15
11	733	1. NMEA	46.78	18.12
		2. WLS	62.33	37.96
		2. 3DMA GNSS	36.85	28.13
		3. LC-FGO	37.82	27.85
		4. LC-FGO-PP	32.13	26.08

**Table 2:** Statistics of vehicle-mounted trip results.

Algorithm	RMSE (m)	STD (m)
1. WLS	14.92	9.20
2. 3DMA GNSS	7.94	4.85
3. LC-FGO	8.09	4.55
4. LC-FGO-PP	5.80	2.95

**Figure A1:**
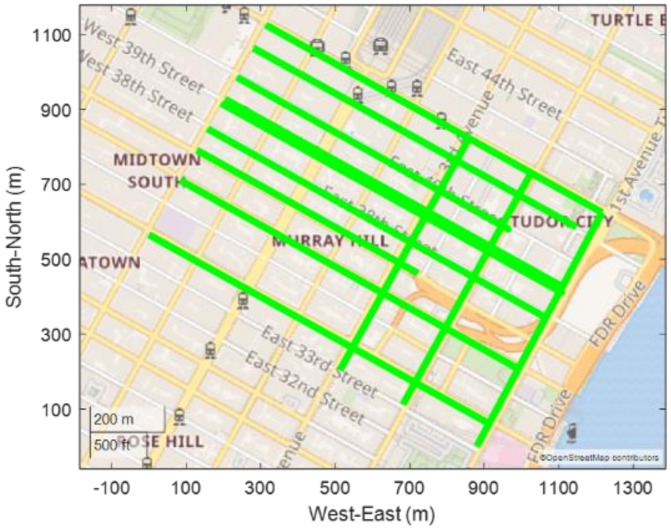
Map plot of a total of 11 experimental navigation trips in New York, each straight green line represents one trip, correspondingly.

**Figure A2:**
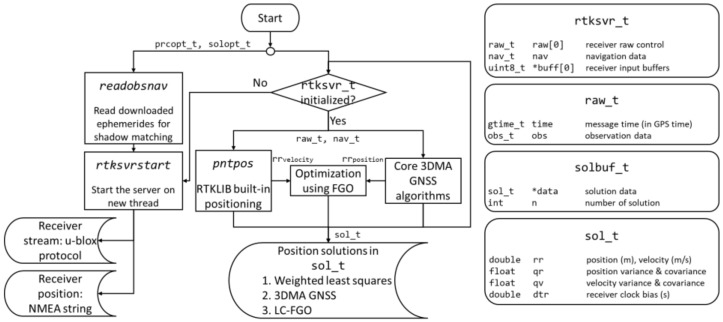
Program flow of this study. The left part is the program structure. The right part is the data dictionary of the primary data structure and members used for implementation.

## Data Availability

Not applicable.

## Appendix A

This appendix presents the map plot of a total of 11 experimental navigation trips in New York City, as shown in Figure A1.

## Appendix B

This appendix presents the main modified functions and IO from RTKLIB. The development environment is under ubuntu 18.04. RTKLIB 2.4.3 b34 is used.
